# Efficacy of root canal treatment for autotransplanted third molars: a 6-Year cohort study of 167 teeth in southern China

**DOI:** 10.7717/peerj.18824

**Published:** 2025-01-09

**Authors:** Bangfeng Han, Liu Liu, Zhishen Jiang, Li Ye, Yubin Cao, Jian Pan

**Affiliations:** 1 Shenzhen Stomatological Hospital, Southern Medical University, Shenzhen, China; 2 State Key Laboratory of Oral Diseases & National Center for Stomatology & National Clinical Research Center for Oral Diseases, West China Hospital of Stomatology, Sichuan University, Chengdu, China; 3 Department of Conservative Dentistry and Endodontics, West China Hospital of Stomatology, Sichuan University, Chengdu, China; 4 Department of Oral and Maxillofacial Surgery, West China Hospital of Stomatology, Sichuan University, Chengdu, China; 5 Department of Evidence-based Stomatology, West China Hospital of Stomatology, Sichuan University, Chengdu, China

**Keywords:** Oral surgery, Dental autotransplantation, Impacted teeth, Root canal treatment

## Abstract

**Background:**

Autogenous tooth transplantation offers significant advantages and promising success rates for replacing non-retainable teeth. This study aimed to investigate the prognostic factors, especially the impact of root canal treatment (RCT), of autotransplanted teeth in an up-to-6-year follow-up cohort of 167 teeth in Southern China.

**Methods:**

We enrolled adult patients from the Southern Medical University-Shenzhen Stomatology Hospital between 2017 and 2023. Patients underwent autogenous tooth transplantation to replace non-retainable molars with upper or lower third molars with Moorrees tooth development stage ≥5. All surgical procedures were performed by an experienced surgeon. The included patients were followed up for 6~72 (median 28.5) months. Success, failure, and survival rate and prognostic factors were evaluated using univariable Kaplan-Meier, multivariable generalized linear regression, and multivariable COX regression analyses.

**Results:**

The overall success rate is 97.6% with four unsuccessful cases. Herein, two of them were removed, leading to an overall survival rate of 98.8%. A total of 159 cases (95%) received RCT in 3 months. Univariable log-rank analysis showed that RCT (RR 0.109, 95%CI 0.010 to 1.202, *P* = 0.028) and site relationship between donor and receipt sites (RR 3.359, 95% CI [1.210–9.329], *P* = 0.020) were two significant prognostic factors of autotransplanted teeth. Multivariable generalized linear regression revealed that RCT is the only significant factor protecting the success rate of autotransplanted teeth (HR 0.003, 95% CI [0.000–0.249], *P* = 0.010). However, in the Cox regression model, the effects of RCT (HR 0.009, 95% CI [0.000–2.514], *P* = 0.101) did not reach statistical significance. Other factors did not demonstrate a significant impact in this cohort. These results supported that autogenous tooth transplantation is a viable alternative to conventional implant treatment with strict indications. Our findings underscore the importance of RCT in transplanted teeth with closed or semi-closed apices. Multi-center observational studies with larger sample size and extended follow-up duration may be needed to validate the conclusion.

## Introduction

Autogenous tooth transplantation, defined as the planned surgical movement of a donor tooth within the same patient, represents a therapeutic approach for replacing non-restorable or missing teeth (Tsukiboshi, Tsukiboshi & Levin, 2023). The donor teeth can be embedded or erupted third molars, premolars, canines, and even supernumerary teeth, which are usually less functional in the original position but potential to function effectively in the recipient site. The recipient socket usually undergoes controlled extraction and possibly surgical preparation before transplantation. The indications of autogenous tooth transplantation include replacement of permanent teeth associated with poor prognosis, replacement of developmentally missing teeth, management of alveolar clefts, repositioning impacted or ectopic teeth, management of oroantral communications, autotransplantation of deciduous teeth as space maintainers, and cases of maxillomandibular reconstructions (Al-Khanati, Albassal & Kara Beit, 2022). Despite the maturity of implant rehabilitation, autogenous teeth and roots have irreplaceable advantages in restoring natural periodontium, proprioception, and alveolar bone volume (Terheyden & Wüsthoff, 2015).

Autogenous tooth transplantation typically yields stable transplanted teeth without residual inflammation, facilitating satisfactory mastication functions and eliciting a positive biological response, as evidenced by normal radiographic manifestations (Zufía et al., 2017; Dhar et al., 2022). Recent advancements in diagnostic and surgical techniques have significantly bolstered the success rates of autotransplantation, exceeding 95% in children and adolescents and 80% in adults (Barendregt et al., 2023). Long-term studies have indicated impressive 5-year survival rates ranging between 81% and 98.2%, particularly noteworthy in cases involving teeth with open apices have >95% 5-year and 10-year survival rates (Tan et al., 2023; Sicilia-Pasos et al., 2022). Limited high-level evidence exists on the long-term outcomes of complete root formation, with the survival rate varying from 81.51% to 100% in different studies and showing a critical risk of bias (Huang et al., 2023). Despite these generally encouraging outcomes, prognostic variations, particularly in adults, continue to challenge surgeons and patients alike.

The efficacy of autogenous tooth transplantation hinges on multifactorial considerations, including patient age and sex, tooth morphology and root development, alveolar bone volume and quality, and the compatibility between donor and recipient sites (Suwanapong et al., 2021). Surgical factors such as extraction technique, extraoral time, and stabilization methods can also influence the prognosis (Jang et al., 2016). Additionally, post-transplantation complications, such as inflammation, root resorption, ankylosis, pulp necrosis, and compromised periodontal recovery often impede the survival of transferred teeth (Tan et al., 2023). In this case, measures to improve the long-term prognosis include endodontic therapies (*e.g*., root canal treatment (RCT)), prosthetics of transferred teeth (*e.g*., crown restoration), bone and soft tissue grafting, and orthodontic interventions, all contingent upon diligent patient compliance. Among practitioners, there is debate about whether RCT should be conducted before, during, or after transplantation, with some advocating for it only if clinical or radiographic signs of pulp necrosis appear (Al-Khanati & Kara Beit, 2022). Although several cohorts of autogenous tooth transplantation have been emerging over the past decade, there remains lacking studies of prospective design, large sample quantity, and long follow-up duration in China (Li et al., 2022; Yang, Jung & Pang, 2019).

Therefore, our aim is to establish a large-sample cohort of Southern Chinese adults to elucidate long-term success, survival, and failure rates, and to explore whether compliance with RCT will affect the success of autotransplanted teeth.

## Materials and Methods

### Setting

The Research Ethics Committee of Southern Medical University-Shenzhen Stomatology Hospital approved the study protocol (SMUSSH-2017-0001). Patients were consecutively enrolled at the Southern Medical University-Shenzhen Stomatology Hospital, from December 2017 to June 2023 prospectively. This prospective cohort study adhered to the Strengthening the reporting of observational studies in epidemiology (STROBE) statement.

### Participants

We obtained written informed consent from enrolled participants. The following were the inclusion criteria: (1) patients > 18 years old; (2) patients have at least a first or second molar which should be removed due to caries sequelae, tooth defect, tooth fracture, or periapical lesions; (3) patients have at least a healthy, erupted or partially erupted, upper or lower third molar with Moorrees tooth development stage ≥5 (Moorrees, Fanning & Hunt, 1963). Patients were excluded if they had local lesions (*e.g*., moderate or severe periodontitis, bruxism, and hemangioma) or systemic disease that contraindicated tooth extraction or tooth transplantation (*e.g*., diabetes, uncontrolled hypertension, heart failure) and if the extracted teeth were accompanied with an adjacent cyst, tumor, fracture, and osteomyelitis diagnosed by medical history and clinical and radiographic examinations. All the patients were required to the complete the periodontal therapy before the tooth transplantation, and patients not receiving periodontal therapy were excluded. If the root of the donor tooth fractures and the remaining root length is more than 2/3 of the total root length with sufficient support, the case would be excluded from the cohort. Patients with traumatic extractions, extended extraction times, prolonged transplant extraoral time were also excluded. Patients with less than 6 months of follow-up or lost to follow-up were excluded from the analysis.

### Surgical procedure

**Figure 1:**
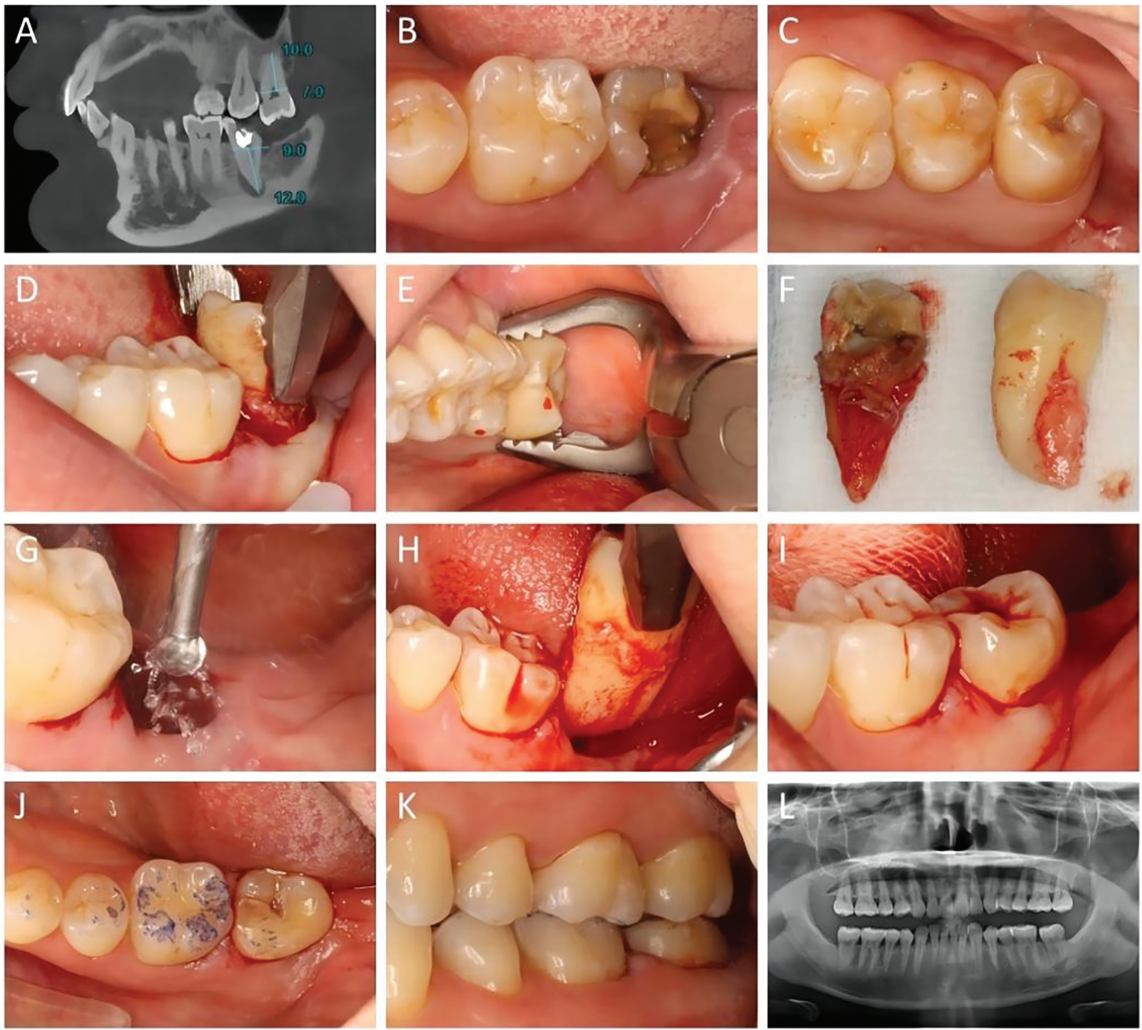
Representative images of the workflow of the autogenous tooth transplantation. (A) Preoperative CBCT imaging of a 31-year-old female. The lower left second molar (37) suffered periapical periodontitis with a poor prognosis. The donor tooth, upper left third molar (28) had root development at stage 5 14, with closed apex and overeruption. Radiographic evaluation indicated a good match between the donor tooth and recipient site. (B) Intra-oral view of the affected 37 at the recipient site. (C) Presentation of the donor tooth 28. (D) Extraction of tooth 37. (E) Extraction of tooth 28. (F) Comparison of the tooth morphology of the recipient and donor teeth. (G) Preparation of the recipient site using a 2.0 mm round bur with saline irrigation. (H) Try-in of the donor tooth. (I) Placement of the donor tooth, possibly fixed with sutures. (J) Occlusion examination showing centric occlusion with light centric contact after transplantation. (K) No interference in lateral occlusion. (L) Immediate panoramic radiograph after the surgery.

All surgical procedures were performed under an nerve block (2% lidocaine with 1/100,000 epinephrine) or infiltration anesthesia (4% articaine with 1/100,000 epinephrine) by a single experienced surgeon (Fig. 1). The surgical steps include: (1) Disinfection and anesthesia. (2) Extraction of the affected tooth: Using standardized surgical instruments and standardized minimally invasive techniques. Teeth hemi-sectioning (root separation) was performed before extraction to decrease the alveolar bone damage. (3) Preparation of the recipient socket. Recipient socket preparation was performed *via* surgical contra-angled handpiece and round carbide bur (20,000 rpm) with copious saline irrigation. (4) Extraction of the donor tooth. Repeat clamping of donor tooth and bone removal around the tooth was avoided to protect the periodontal tissue. (5) Transplantation of the donor tooth. (6) Suturing the mucoperiosteal flap. (7) Occlusal adjustment and fixation. If there is no occlusal contact both medially and laterally, or if there is only medial occlusal contact with no lateral occlusal contact, the teeth would be immobilized with suture fixation; if there is medial and lateral occlusal contact, it was adjusted to light occlusal contact and then fixed elastically with a fibrous splint. (8) Radiographic evaluation. Notably, we store the donor tooth in physiological saline and implant it into the recipient area within 30 min.

### Follow-up timeline

After the operation, we advised the patients to complete the RCT 2 to 4 weeks after the tooth autotransplantation. We conduct routine oral clinical examinations for every patient during their follow-up visits, including percussion, mobility, occlusion, and periodontal probing examinations at 1, 2, 3, 6, 9, 12, 18, and 24 months. We performed the digital dental X-rays at the follow-up of 1 year.

### Data collection

Demographical data (age and gender), donor and receipt site, and reasons for tooth extraction were recorded in the electronic medical record system preoperatively. During the surgery, the surgical assistant recorded whether collagen sponge/matrix was used to fill the socket. Collagen sponge/matrix was used to ensure proper healing and initial support only when the donor tooth had inadequate initial stability in the receipt socket. In addition, whether additional fiber-reinforced composite periodontal splint was used to fix the autotransplanted tooth was also recorded. Moreover, the time of extraoral time was recorded by a stopwatch. During the follow-up period, RCT, prosthetic restoration, and the outcome of autotransplanted teeth were recorded.

### Definition of success, survival, and failure

At least 1 year after the surgery, the transplanted teeth are evaluated for healing according to the criteria to determine if the transplantation is successful. The outcome of autotransplanted tooth was assessed by an independent dentist. Any complications or undesired outcome were notified to the surgeon.

The success criteria for transplantation of fully developed teeth are healing of the gums, periodontal ligament, and alveolar bone, and obtaining proper root canal treatment. Radiographic examinations show a normal width of the periodontal ligament space around the transplanted tooth, no signs of progressive root resorption, and no periapical or periodontal lucency. Clinical examination shows tooth mobility within the normal physiological range, normal percussion sound, no signs of attachment loss (no formation of periodontal pockets), no signs of inflammation, no discomfort, and the ability to perform normal tooth function. Success rate of autogenous tooth transplantation refers to the proportion of successfully transplanted teeth that meet the criteria for successful transplantation out of the total number of transplanted teeth.

Survival refers to not only successfully transplanted teeth but also partially functional transplanted teeth with grade I-II looseness, transplanted teeth with abnormal percussion sound, and transplanted teeth with root-alveolar bone adhesion. Failure refers transplanted teeth that have fallen off, or have grade III looseness, or have inflamed periodontal tissues and cannot function properly, or X-ray examination shows significant root absorption or significant absorption of the surrounding bone tissues.

### Statistical analysis

For univariable analysis, the Kaplan-Meier curve and the Mantel-Haenszel method were used to calculate the risk ratio (RR) with 95% confidence intervals (CI). Herein, we calculated coefficient for continuous data and transformed coefficient to RR in the tables. For multivariable analysis, the dichotomous data (success rate) was analyzed by the generalized linear regression model, and RR with 95% CI was estimated. Then, the time-to-event success data was analyzed by multivariable Cox models, and hazard ratio (HR) with 95% CI was estimated. Analysis was performed using STATA (version 14.0, StataCorp LLC, TX, USA). *P* < 0.05 was considered as statistically significant.

## Results

The patients’ characteristics and treatment is provided in Table 1. This study included a cohort of 167 autotransplanted teeth in 166 participants (120 women and 46 men), aged between 18 and 60 years old. Among the 167 autotransplanted teeth, 94 (56%) of them received RCT within 2 to 4 weeks, 89% completed it within 2 months, and the 160 cases (96%) received RCT in 3 months.

**Table 1:** Description of patients’ characteristics and treatment.

	N/mean	%/range
Gender		
Female	120	72.3
Male	46	27.7
Age (y)	29	18–60
Follow-up (m)	28.5	6.3–72
Donor site		
18	43	25.3
28	39	23.5
38	47	28.3
48	38	22.9
Recipient site		
16	10	5.4
17	13	7.8
26	8	4.8
27	15	9.0
36	41	24.7
37	23	13.9
46	35	21.1
47	22	13.3
Site relationship		
sSsJ	96	57.2
sSoJ	28	16.9
oSsJ	20	12.0
oSoJ	23	13.9
Extraction reason		
Caries and sequelae	112	66.9
Periapical lesion	34	20.5
Tooth fracture	17	10.2
External resorption	4	2.4
Fixation		
No	66	39.8
Yes	101	66.2
Collagen sponge		
No	117	69.9
Yes	50	30.1
RCT		
No	7	4.2
Yes	160	84.3
Prosthetic restoration		
No	152	91
Yes	15	9
Extraoral time (min)	17.6	6.3–28.7

**Notes:**

Number and percentages were presented for dichotomous or categorical variables, whereas mean and range were presented for continuous variables.

Abbreviations: sSsJ, donor and recipient teeth are located on the same side of the same jaws; sSoJ, donor teeth are located on the lateral side of the same jaws of the recipient teeth; oSsJ, donor teeth are located on the same side of the opposite jaws of the recipient teeth; oSoJ, donor teeth are located on the opposite side of the opposite jaws of the recipient teeth; RCT, root canal treatment.

**Figure 2:**
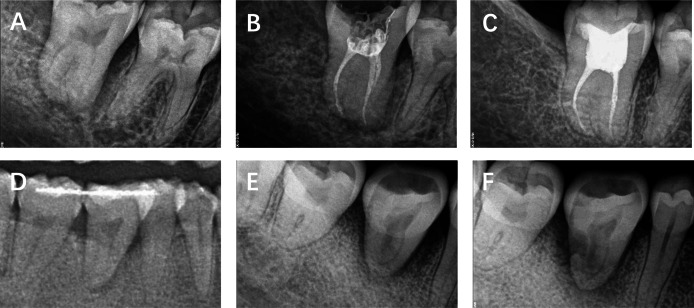
The radiographic images of representative cases. (A–C) Case 1. (A) Immediately postoperative. (B) Root filling completion at postoperative 4 weeks. (C) Follow-up at postoperative 24 months. (D and E) Case 2. (D) Immediately postoperative. (E) Rejecting RCT treatment at postoperative 8 weeks. (F) Follow-up at postoperative 28 months.

After a median follow-up of 28.5 months, the overall success rate is 97.6% with four unsuccessful cases. Herein, 2 of them were removed, leading to an overall survival rate of 98.8%. Details of these unsuccessful cases (including one failure case) were presented in Table 2. The representative of successful and failed cases were presented below (Fig. 2):

**Table 2:** Details of unsuccessful cases.

No.	Gender	Age	Follow-up (y)	Donor site	Receipt site	Extral-oral time (min)	RCT	Reason	Treatment
1	Female	27	2.18	18	16	17.0	No	Grade III tooth mobility	Removal
2	Female	34	2.36	38	46	13.7	No	Internal tooth resorption	RCT
3	Female	24	2.56	28	46	9.3	Yes	Periapical lesion	Root canal retreatment
4	Male	28	2.93	18	27	20.5	No	Grade III tooth mobility	Removal

Case 1, female, 25y, presented to our department 5 years ago due to tooth defect of 47 (Figs. 2A–2C). We transplanted 48 to the site of 47. The participant completed the RCT treatment in 2 to 4 weeks after the autotransplantation. After 2 years post-operation, the autotranplanted tooth remains asymptomatic, with a clear periodontal membrane, a normal trabecular pattern of the alveolar bone, and without root-bone adhesion.

Case 2, female, 34y, presented to our department 5 years ago due to tooth defect of 46 (Figs. 2D and 2E). We transplanted 38 to the site of 46. The participant revisited us at postoperative 2 months but rejected the RCT treatment. After 28 months post-operation, the patient revisited us due to a loose autotranplanted tooth. The transplanted tooth exhibits grade 3 looseness, and there is internal resorption in the mesial root with a periapical lucency. The tooth was removed.

**Figure 3:**
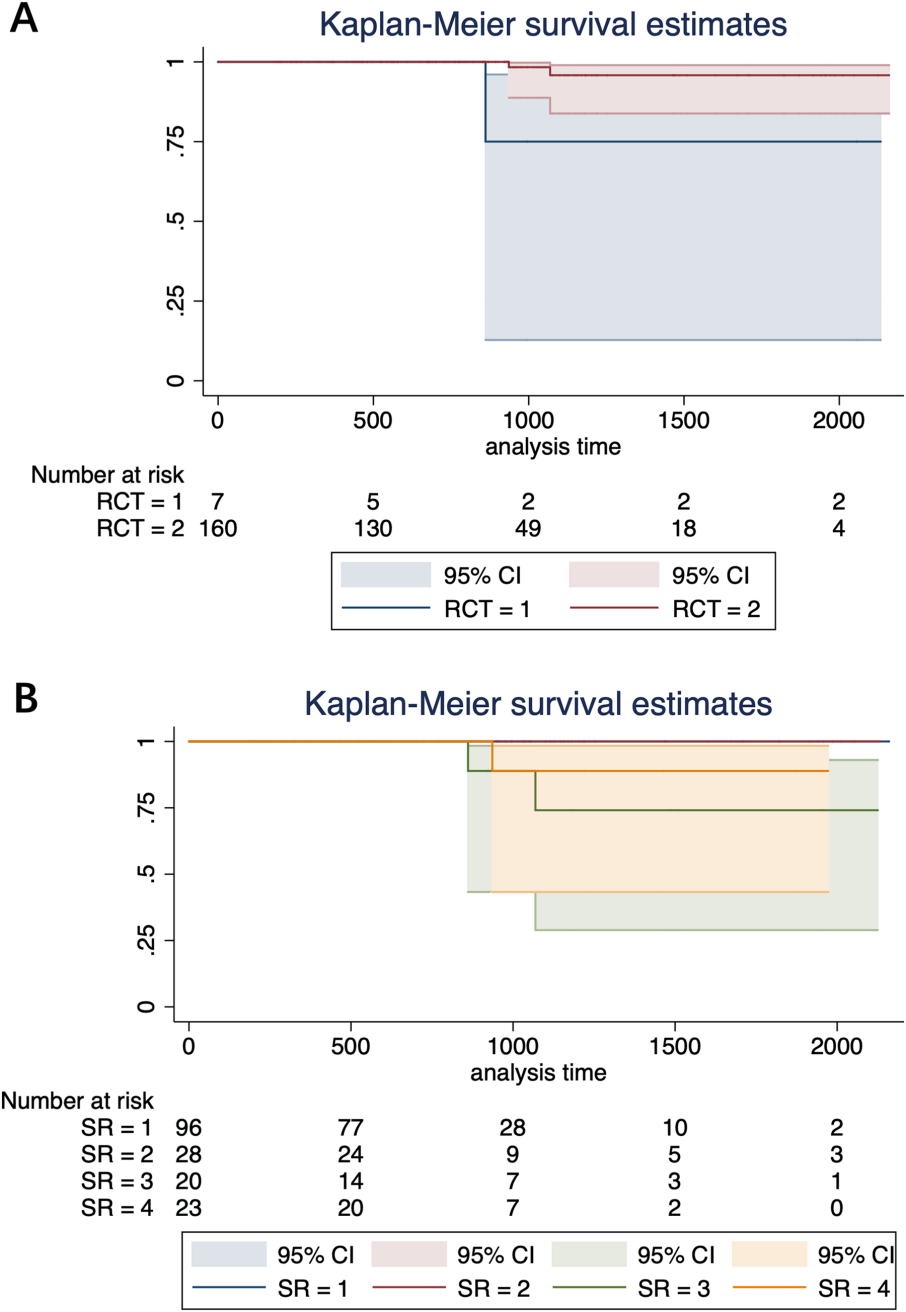
The cumulative failure rate of RCT received by the patients with autotransplanted tooth. (A) Grouped by RCT. RCT = 1, without treatment; RCT = 2, with treatment. (B) Grouped by site relationship. SR = 1, donor and recipient teeth are located on the same side of the same jaws; SR = 2, donor teeth are located on the lateral side of the same jaws of the recipient teeth; SR = 3, donor teeth are located on the lateral side of the same jaws of the recipient teeth; SR = 4, donor teeth are located on the opposite side of the opposite jaws of the recipient teeth.

In univariable analysis, two significant factors affect the prognosis of autotransplanted teeth. RCT (RR 0.109, 95% CI [0.010–1.202], *P* = 0.028) and site relationship between donor and receipt sites (RR 3.359, 95% CI [1.210–9.329], *P* = 0.020) (Fig. 3). However, we did find significant effects of RCT timing (within 1 month or between 1 to 3 months) on the prognosis. In the generalized linear regression, RCT is the only significant factor protecting the success rate of autotransplanted teeth (HR 0.003, 95% CI [0.000–0.249], *P* = 0.010). In contrast, site relationship between donor and receipt sites (HR 7.103, 95% CI [0.792–63.742], *P* = 0.080) did not reach statistical significance (Table 3). In the Cox regression model, both the effects of RCT (HR 0.009, 95% CI [0.000–2.514], *P* = 0.101) and the site relationship of donor and receipt sites (HR 6.950, 95% CI [0.483–100.069], *P* = 0.154) did not reach statistical significance. Other factors were statistically insignificant in all the univariable and multivariable models (Table 4).

**Table 3:** Multivariable generalized linear regression assessing the prognostic factors of success of autotransplanted teeth.

	Risk ratio	95% LCI	95% UCI	*P*
Gender	0.407	0.024	6.892	0.533
Age	1.001	0.807	1.243	0.992
Donor	0.988	0.872	1.119	0.847
Receipt	1.000	0.855	1.171	0.996
Site relationship	7.103	0.792	63.742	0.080
Collagen sponge	2.759	0.164	46.401	0.481
RCT	0.003	0.000	0.249	0.010
Extraoral time	1.074	0.782	1.477	0.659

**Table 4:** Multivariable Cox regression analysis assessing the prognostic factors of success of autotransplanted teeth.

	Hazard ratio	95% LCI	95% UCI	*P*
Gender	1.342	0.028	64.627	0.882
Age	0.998	0.736	1.352	0.988
Donor	0.993	0.818	1.206	0.945
Receipt	0.971	0.789	1.194	0.779
Site relationship	6.950	0.483	100.069	0.154
Collagen sponge	4.987	0.176	141.174	0.346
RCT	0.009	0.000	2.514	0.101
Extraoral time	1.001	0.630	1.592	0.996

## Discussion

In this 6-year study, we conducted autogenous tooth transplantation in 167 teeth in Southern China with a mean follow-up of 28.5 months. The overall success rate is 97.6%, with four unsuccessful cases. Among the unsuccessful cases, two teeth were removed due to irreversible Grade III tooth mobility, while the remaining two underwent root canal (re)treatment for salvage, resulting in an overall survival rate of 99.4%. Our findings highlight the importance of timely RCT after autotransplantation, particularly within 3 months, as evidenced by the outcomes of transplanted teeth. The variables in patient factors (gender and age) and surgical factors (fixation and collagen) did not show a significant impact in the present study. In addition, prosthetic restoration may also be a potential factor influencing the survival of autotransplanted teeth.

All teeth included in our study had complete root formation (Moorrees stage > 4) but ranging in apical foramen closure, and we carried out RCT as a normal treatment procedure. Existing literature suggests the consensus that RCT is essential for the survival of transplanted teeth with closed apex or complete root formation, especially in adults (Lin et al., 2020). As to teeth with open apex, the choice tends to lean towards monitoring pulp vitality after transplantation and allowing for potential pulpal healing (Armstrong, O’Reilly & Ahmed, 2020). Although we did not distinguish open or closed apex, our outcomes strongly indicate that RCT could be a key procedure contributing to the autotransplanted teeth with Moorrees stage > 4 in adult patients (age > 18y). Interestingly, in Yu et al. (2017), which included transplanted third molars with completely formed roots in patients over 18 years, RCT was procedurally implemented only on those older than 20 years, and the overall survival rate was 90.8%. Another retrospective study revealed that of the teeth that were followed for at least 5 years, 59.3% had complete root formation with no signs of pathology and required no RCT (Murtadha & Kwok, 2017). Therefore, despite the potential for pulpal revascularization in transplanted teeth with an open apex, our results cautiously underscore the importance of preventive RCT following autotransplantation in every adult patient.

Patients’ compliance to RCT has been shown to affect the prognosis of transplanted teeth significantly, as evidenced in our study. In our research, eight cases declined preventive RCT after autotransplantation, two of them ultimately being removed. Conversely, among the other 159 cases that underwent RCT within 3 months, all survived, with only one requiring retreatment. Regarding the timing of RCT, the American Association of Endodontists recommends extirpating pulp tissue from autotransplanted teeth with closed apices within 7 to 14 days post-surgery (Cohen, Shen & Pogrel, 1995). Chung et al. (2014) reported comparable failure rates (Incidence Risk Ratio 1.0, 95% CI [0.2–5.2]) between groups receiving RCT pre- or within 14 days post-transplant and those receiving it more than 14 days post-transplant. However, they noted a doubled incidence of infection-related root resorption (incidence risk ratio 2.0, 95% CI [0.2–9.3]) in the latter group. Another population-based study found no significant difference in survival probability among autotransplanted teeth receiving postoperative RCT at various intervals (*i.e*., <2 weeks, 2–4 weeks, 4–8 weeks, and >8 weeks after transplantation) (Lin et al., 2020). Overall, the timing of RCT after transplantation remains a topic of debate. However, our study cautiously suggests that prompt return for preventive RCT and timely therapeutic RCT contribute to the survival of transplanted teeth.

In addition to the surgery and endodontic treatment, achieving a favorable long-term prognosis for autogenous tooth transplantation requires close collaboration among various dental specialists, including prosthodontists and orthodontists. While prosthetic restoration did not emerge as a significant protective factor in our survival and univariate analyses, its impact warrants attention. Indeed, the clinical experience appears to be that following effective RCT, prosthetic restoration becomes necessary to prevent complications and restore both morphology and function to transplanted teeth. However, previous research has paid limited attention to the prognostic influence of prosthetic treatment in autotransplanted third molars. Studies on prosthetic treatment in the autotransplantation of premolars to the anterior maxilla suggest that in young teeth, permanent restoration should occur after complete root formation to mitigate the risk of complications (Akhlef et al., 2018). Additionally, although the present study regretfully did not involve orthodontic treatment, reports indicate that early application of orthodontic force may enhance the success rate of autotransplanted teeth with complete root formation. This effect could be attributed to the reduction of ankylosis and promotion of periodontal tissue regeneration (Kokai et al., 2015).

Considering the influence of post-operative procedures on the success and survival of autotransplanted teeth, we can underscore the importance of patient adherence after autogenous tooth transplantation from the present study. Patient compliance with post-operative protocols can be influenced by various factors, including socioeconomic status, individual expectations and anxieties, quality of doctor-patient communication, and clinical techniques employed (Aspegren, 1999). In addition to providing proper medical instructions and post-surgical health education, it’s important to recognize the role of patient-reported outcomes (PROs) in promoting internal motivation for adherence (Wang & Gottumukkala, 2021). PROs serve two key purposes: alleviating the burden of disease and enhancing patient-centered care (Black, 2013). On one hand, patients are usually affected by their symptoms not only biologically, such as the pain and swelling pre-, intra-, and post-treatment, but also psychologically, such as tension, fear, anxiety, and even depression. Addressing these burdens through effective pain management, timely administration of preventive medications, and supportive care is essential (Jaensson, Dahlberg & Nilsson, 2019). On the other hand, patient-reported experience measures, associated with patient satisfaction, personal costs, expectation fulfillment, and decision regrets, can be influenced by many clinical and nonclinical factors, including the dentist’s clinical expertise and interpersonal skills (Doğramacı & Rossi-Fedele, 2023). Adopting a more patient-centered approach that aligns the expectations of both dentists and patients, for instance, by prioritizing both functional and aesthetic outcomes, can significantly enhance patient adherence and satisfaction (Zhang et al., 2022).

The present study has several limitations that warrant cautious interpretation of the results. Firstly, detailed clinical parameters related to surgical procedures, such as extraoral times, fixation periods, antibiotic usage, or bone grafting, were not included. Second, we preserved the donor tooth in saline according to the Chinese expert consensus; however, Hank’s balanced salt solution and coconut water are the best storage media (Ustad et al., 2013). Third, the longevity of autogenous tooth transplants, is significantly affected by various factors, including general health, oral health status, smoking status, the level of care provided to these teeth, and the use of different oral hygiene practices and health behaviors. A study collecting more comprehensive covariates may be needed in the future. Forth, novel techniques may be adopted in future studies, such as piezosurgery and 3D-printed tooth replicas, to further improve the outcome of autotransplanted teeth. Fifth, further subdivision into subgroups is necessary to better understand the influence of postoperative treatment, in a cohort with a larger sample size and extended follow-up duration.

## Conclusions

In conclusion, our study suggests that performing RCT in transplanted teeth significantly correlates with the success of autotransplantation in adult patients. Compliance with RCT should be emphasized and monitored for these transplanted teeth with closed apices. Proper postoperative management, coupled with high patient compliance, may contribute to favorable long-term outcomes and survival of transplanted teeth. The findings regarding success and survival rates support the feasibility of using autotransplantation to replace non-retainable first or second molars with third molars, serving as a viable alternative to conventional implant treatment when appropriate indications are observed. A cohort with larger sample size, extended follow-up duration, and more comprehensively collected covariates may be needed to draw the more robust conclusion.

## Supplemental Information

10.7717/peerj.18824/supp-1 Supplemental Information 1 Raw data and codebook.

10.7717/peerj.18824/supp-2 Supplemental Information 2 STROBE checklist.

## Additional Information and Declarations

### Data Availability

The following information was supplied regarding data availability: The code and data are available in the Supplemental File.

## References

[ref-1] Akhlef Y, Schwartz O, Andreasen JO, Jensen SS (2018). Autotransplantation of teeth to the anterior maxilla: a systematic review of survival and success, aesthetic presentation and patient-reported outcome. Dental Traumatology.

[ref-2] Al-Khanati NM, Albassal A, Kara Beit Z (2022). Unusual indications of teeth transplantation: a literature review. Cureus.

[ref-3] Al-Khanati NM, Kara Beit Z (2022). Reconsidering some standards in immediate autotransplantation of teeth: case report with 2-year follow-up. Annals of Medicine & Surgery.

[ref-4] Armstrong L, O’Reilly C, Ahmed B (2020). Autotransplantation of third molars: a literature review and preliminary protocols. British Dental Journal.

[ref-5] Aspegren K (1999). BEME Guide No. 2: teaching and learning communication skills in medicine-a review with quality grading of articles. Medical Teacher.

[ref-6] Barendregt D, Andreasen JO, Leunisse M, Eggink E, Linssen M, Van der Weijden F, Louropoulou A (2023). An evaluation of 1654 premolars transplanted in the posterior region-A retrospective analysis of survival, success and complications. Dental Traumatology.

[ref-7] Black N (2013). Patient reported outcome measures could help transform healthcare. BMJ.

[ref-8] Chung WC, Tu YK, Lin YH, Lu HK (2014). Outcomes of autotransplanted teeth with complete root formation: a systematic review and meta-analysis. Journal of Clinical Periodontololy.

[ref-9] Cohen AS, Shen TC, Pogrel MA (1995). Transplanting teeth successfully: autografts and allografts that work. Journal of American Dental Association.

[ref-10] Dhar S, Singh G, Mishra M, Gaur A (2022). A prospective study on autotransplantation of mandibular third molars with complete root formation. Craniomaxillofacial Trauma & Reconstruction.

[ref-11] Doğramacı EJ, Rossi-Fedele G (2023). Patient-related outcomes and oral health-related quality of life in endodontics. International Endodontic Journal.

[ref-12] Huang J, Gan Y, Han S, Xu HE, Yuan YI, Zhu HE, Tian X, Li N, Li D, Cai Z (2023). Outcomes of autotransplanted third molars with complete root formation: a systemic review and meta-analysis. Journal of Evidence-Based Dental Practice.

[ref-13] Jaensson M, Dahlberg K, Nilsson U (2019). Factors influencing day surgery patients’ quality of postoperative recovery and satisfaction with recovery: a narrative review. Perioperative Medicine.

[ref-14] Jang Y, Choi YJ, Lee SJ, Roh BD, Park SH, Kim E (2016). Prognostic factors for clinical outcomes in autotransplantation of teeth with complete root formation: survival analysis for up to 12 years. Journal of Endodontics.

[ref-15] Kokai S, Kanno Z, Koike S, Uesugi S, Takahashi Y, Ono T, Soma K (2015). Retrospective study of 100 autotransplanted teeth with complete root formation and subsequent orthodontic treatment. American Journal of Orthodontics And Dentofacial Orthopedics.

[ref-16] Li YQ, Hui XY, Xu GJ, Ma YY, Yang X, Xu J, Zhu QL, Zhang ZM, Wu X, Hou R (2022). Screening and analysis of prognostic factors of repairing single missing tooth by autotransplantation of teeth. Zhonghua Kou Qiang Yi Xue Za Zhi.

[ref-17] Lin PY, Chiang YC, Hsu LY, Chang HJ, Chi LY (2020). Endodontic considerations of survival rate for autotransplanted third molars: a nationwide population-based study. International Endodontic Journal.

[ref-18] Moorrees CF, Fanning EA, Hunt EE (1963). Age variation of formation stages for ten permanent teeth. Journal of Dental Research.

[ref-19] Murtadha L, Kwok J (2017). Do autotransplanted teeth require elective root canal therapy? A long-term follow-up case series. Journal of Oral & Maxillofacial Surgery.

[ref-20] Sicilia-Pasos J, Kewalramani N, Peña-Cardelles JF, Salgado-Peralvo AO, Madrigal-Martínez-Pereda C, López-Carpintero Á (2022). Autotransplantation of teeth with incomplete root formation: systematic review and meta-analysis. Clinical Oral Investigation.

[ref-21] Suwanapong T, Waikakul A, Boonsiriseth K, Ruangsawasdi N (2021). Pre- and peri-operative factors influence autogenous tooth transplantation healing in insufficient bone sites. BMC Oral Health.

[ref-22] Tan BL, Tong HJ, Narashimhan S, Banihani A, Nazzal H, Duggal MS (2023). Tooth autotransplantation: an umbrella review. Dental Traumatology.

[ref-23] Terheyden H, Wüsthoff F (2015). Occlusal rehabilitation in patients with congenitally missing teeth-dental implants, conventional prosthetics, tooth autotransplants, and preservation of deciduous teeth-a systematic review. International Journal of Implant Dentistry.

[ref-24] Tsukiboshi M, Tsukiboshi C, Levin L (2023). A step-by step guide for autotransplantation of teeth. Dental Traumatology.

[ref-25] Ustad F, Ali FM, Kota Z, Mustafa A, Khan MI (2013). Autotransplantation of teeth: a review. American Journal of Medical and Dental Sciences.

[ref-26] Wang XS, Gottumukkala V (2021). Patient-reported outcomes: is this the missing link in patient-centered perioperative care?. Best Practice & Research-Clinical Anaesthesiology.

[ref-27] Yang S, Jung BY, Pang NS (2019). Outcomes of autotransplanted teeth and prognostic factors: a 10-year retrospective study. Clinical Oral Investigations.

[ref-28] Yu HJ, Jia P, Lv Z, Qiu LX (2017). Autotransplantation of third molars with completely formed roots into surgically created sockets and fresh extraction sockets: a 10-year comparative study. International Journal of Oral & Maxillofacial Surgery.

[ref-29] Zhang CN, Zhu Y, Zhang YJ, Jiang YH (2022). Clinical esthetic comparison between monolithic high-translucency multilayer zirconia and traditional veneered zirconia for single implant restoration in maxillary esthetic areas: prosthetic and patient-centered outcomes. Journal of Dental Sciences.

[ref-30] Zufía J, Abella F, Trebol I, Gómez-Meda R (2017). Autotransplantation of mandibular third molar with buccal cortical plate to replace vertically fractured mandibular second molar: a novel technique. Journal of Endodontics.

